# A Biodegradable Mg-Based Alloy Inhibited the Inflammatory Response of THP-1 Cell-Derived Macrophages Through the TRPM7–PI3K–AKT1 Signaling Axis

**DOI:** 10.3389/fimmu.2019.02798

**Published:** 2019-12-03

**Authors:** Liang Jin, Chenxin Chen, Yutong Li, Feng Yuan, Ruolan Gong, Jing Wu, Hua Zhang, Bin Kang, Guangyin Yuan, Hui Zeng, Tongxin Chen

**Affiliations:** ^1^School of Biomedical Engineering, Med-X Research Institute, Shanghai Jiao Tong University, Shanghai, China; ^2^State Key Laboratory of Metal Matrix Composite, National Engineering Research Center of Light Alloy Net Forming, Shanghai Jiao Tong University, Shanghai, China; ^3^Division of Immunology, Shanghai Children's Medical Center, Institute of Pediatric Translational Medicine, Shanghai Jiao Tong University School of Medicine, Shanghai, China; ^4^Department of Allergy and Immunology, Shanghai Children's Medical Center, Shanghai Jiao Tong University School of Medicine, Shanghai, China; ^5^Department of Orthopaedics, Peking University Shenzhen Hospital of Medicine, Shenzhen, China

**Keywords:** JDBM alloy, macrophages, TRPM7, toll-like receptor 4, magnesium ion

## Abstract

Mg-based alloys might be ideal biomaterials in clinical applications owing to favorable mechanical properties, biodegradability, biocompatibility, and especially their anti-inflammatory properties. However, the precise signaling mechanism underlying the inhibition of inflammation by Mg-based alloys has not been elucidated. Here, we investigated the effects of a Mg-2.1Nd-0.2Zn-0.5Zr alloy (denoted as JDBM) on lipopolysaccharide (LPS)-induced macrophages. THP-1 cell-derived macrophages were cultured on JDBM, Ti−6Al−4V alloy (Ti), 15% extract of JDBM, and 7.5 mM of MgCl_2_ for 1 h before the addition of LPS for an indicated time; the experiments included negative and positive controls. Our results showed JDBM, extract, and MgCl_2_ could decrease LPS-induced tumor necrosis factor (TNF) and interleukin (IL)-6 expression. However, there were no morphologic changes in macrophages on Ti or JDBM. Mechanically, extract and MgCl_2_ downregulated the expression of toll-like receptor (TLR)-4 and MYD88 compared with the positive control and inhibited LPS-induced nuclear factor-kappa B (NF-κB) and mitogen-activated protein kinase (MAPK) signaling pathways by inactivation of the phosphorylation of IKK-α/β, IKβ-α, P65, P38, and JNK. Additionally, the LPS-induced reactive oxygen species (ROS) expression was also decreased by extract and MgCl_2_. Interestingly, the expression of LPS-induced TNF and IL-6 could be recovered by knocking down TRPM7 of macrophages, in the presence of extract or MgCl_2_. Mechanically, the activities of AKT and AKT1 were increased by extract or MgCl_2_ with LPS and were blocked by a PI3K inhibitor, whereas siRNA TRPM7 inhibited only AKT1. Together, our results demonstrated the degradation products of Mg-based alloy, especially magnesium, and resolved inflammation by activation of the TRPM7–PI3K–AKT1 signaling pathway, which may be a potential advantage or target to promote biodegradable Mg-based alloy applications.

## Introduction

Biodegradable Mg-based alloy is a new-generation biomaterial used in cardiovascular stents, orthopedic implants, bone screws, etc. (1–3). Although it can surmount the drawbacks of permanent metallic biomaterials, such as chronic inflammation, in-stent restenosis, and second surgery (4, 5), the rapid corrosion of magnesium matrix not only results in loss of their own structural strength ahead of tissue repair but also greatly alters the implantation microenvironment, with many unexpected influences locally (6, 7). For instance, previous data showed that extract of a Mg-based alloy effectively promoted the proliferation of mouse fibroblasts by regulating the cell cycle, energy metabolism, and protein synthesis and obviously enhanced human mesenchymal stem cells toward osteoblastic differentiation by intricate cellular mechanisms (8, 9). Thus, it is essential to disclose the relationship between cells and biodegradable products of Mg-based alloys.

The foreign body response (FBR) to biomaterial implantation is a critical factor in determining the eventual outcome of surgery (10). Unlike non-biodegradable materials eventually encapsulated with fiber that elicit a lifelong chronic inflammation according to the classical FBR theory (10, 11), biodegradable biomaterials can avoid these adverse effects because of their completely degradable nature. However, the degradation products will also affect the early stage of FBR and bring about unpredictable events. Recently, Cipriano et al. reported that endothelial cells (ECs) will produce a pro-inflammatory cytokine culture with a Mg–Zn–Sr alloy, and Zhou et al. found that extract of a Mg-based alloy converted contractile vascular smooth muscle cells (VSMCs) to an inflammatory phenotype (12, 13). Contradictorily, Rochelson et al. initially showed that magnesium inhibited inflammatory responses of human umbilical vein ECs (HuVECs), and Shechter et al. also reported that oral magnesium could help patients with coronary artery disease by improvement of EC function (14, 15). In addition, magnesium shows an anti-inflammatory effect that is used to treat seizure prophylaxis or cerebral palsy (16, 17). Li et al. reported that magnesium-doped titanium exerted an anti-inflammatory phenotype macrophage (18). Thus, fully understanding the effects of potential molecular mechanism of degradable products of Mg-based alloys on macrophages is essential.

Toll-like receptors (TLRs), such as TLR-2, TLR-4, or TLR-7, are typical pattern recognition receptors (PRRs) of immune cells that sense damage-associated molecular patterns (DAMPs) or pattern-associated molecular patterns (PAMPs) to initiate the innate immune response during the FBR (19, 20). There are reports of the impact of Mg-based alloys on TLRs. Recently, Xia et al. showed that high-purity Mg staples can suppress TLR-4/nuclear factor-kappa B (NF-κB) and activate vascular endothelial growth factor (VEGF) to inhibit inflammation in the rectal anastomoses of mice (21). In addition, Zhai et al. showed that metallic magnesium degradation products inhibit osteoclast differentiation by attenuation of the NF-κB and NFACT1 signaling pathway (22). Nevertheless, the systemic mechanism of the interaction between degradable products of Mg-based alloy and TLRs signaling pathways on macrophages requires further elucidation.

The transient receptor potential cation channel subfamily M, member 7 (TRPM7) is a very ubiquitous cation channel with a fused alpha-kinase domain expressed on the surface that is highly permeable to magnesium and calcium, which regulates cellular physiological metabolism, such as cell proliferation and migration (23). For example, TRPM7 can mediate oxidative and cell morphology change through m-calpain activity (24). Most recently, Zhang et al. found that regulation of TRPM7 by magnesium can enhance the osteoinduction of human osteoblasts by activating the phosphatidylinositol 3-kinase (PI3K) signaling pathway, which encouraged us to explore whether TRPM7 also has an important role in the inflammatory regulation of immune cells during Mg-based alloy implantation (25).

The aim of this study was to disclose potential anti-inflammatory mechanisms of the degradation products of a Mg-based alloy [Mg–Nd–Zn–Zr alloy [JDBM] used as a cardiovascular stent (26)] to THP-1 cell-derived macrophages stimulated by lipopolysaccharide (LPS), a frequently used agent to mimic infectious circumstance (27). We here investigated the expression of pro-inflammatory cytokine tumor necrosis factor-α (TNF-α) and interleukin (IL)-6 of macrophages on the JDBM, Ti−6Al−4V (Ti, used in permanent metallic biomaterials) as the control group, as well as extract of JDBM and MgCl_2_, respectively. Furthermore, the correlating proteins downstream of TLR-4 pathways such as NF-κB and mitogen-activated protein kinase (MAPK), reactive oxygen species (ROS), and the TRPM7–PI3K pathway were analyzed to elucidate potential mechanisms.

## Materials and Methods

### Materials Preparation

The details of composition and the ingot of Mg−2.1Nd−0.2Zn−0.5Zr (wt%, abbreviated as JDBM) used in this study were described in our previous studies (28, 29). Discs of JDBM and Ti with a diameter of 14 mm and height of 2.0 mm were ultrasonically cleaned by ethanol and acetone for 10 min and were further sterilized by exposure to ultraviolet light for another hour. JDBM samples were precorroded in Roswell Park Memorial Institute (RPMI) 1640 culture medium (Gibco, USA) supplemented with 10% inactivated fetal bovine serum (FBS) and 1% penicillin–streptomycin (PS) for 24 h (at 5% CO_2_ and 37°C) to avoid the initial high corrosion and ensure cell adhesion (30). Ti discs and cell culture plates (CCPs) underwent the same pretreatment. Because protein adsorption is an important event during the FBR, the protein concentration of all the soaking solutions described above were analyzed by an indirect evaluation of protein absorption, the bicinchoninic acid (BCA) assay, according to the protocol (19). Extract from JDBM was prepared according to ISO-10993 guidelines. Briefly, disc samples were immersed in RPMI 1640 cell culture medium according to the surface area/volume ratio of 1.25 cm^2^/ml for 72 h at 5% CO_2_ and 37°C. After that, the original JDBM extract was harvested and filtered (0.22 μm). Wang et al. recommended that a minimum of six times to a maximum of 10 times dilution of extract was appropriate to be used in *in vitro* tests because the dilution would not result in cytotoxicity (31). Additionally, in our previous study, we found that 10–20% extract of JDBM could inhibit LPS-induced inflammation (32). Therefore, to explore possible anti-inflammatory effects, the extract was further diluted into 15% extract with cell culture medium as the experiment group (extract). In human body fluid, Mg-based alloy degraded as Mg + H_2_O – Mg(OH)_2_ + H_2_ and then Mg(OH)_2_ + 2Cl^−^ – MgCl_2_ + 2OH^−^ (33), indicating that the MgCl_2_ was the major final metabolic compound of Mg-based alloy in the body, and MgCl_2_·6H_2_O, therefore, was diluted into cell culture medium at 7.5 mM of (180 mg/L) final magnesium ion concentration similar to that of 15% extract.

### Cell Preparation and Treatment

Here, we selected THP-1 cell-derived macrophages because of their strong similarity to human primary macrophages and low cost (34, 35). The THP-1 cell line was obtained from Cell Bank, Shanghai Institutes for Biological Sciences, Chinese Academy of Sciences, Shanghai, China, and was cultured in RPMI 1640 medium supplemented with 10% FBS and 1% PS. THP-1 cells were treated with 50 ng/ml phorbol 12-myristate 13-acetate (PMA; Sigma, USA) for 48 h in order to differentiate into THP-1-derived macrophages and then were refreshed with RPMI 1640 for another 24 h. After that, macrophages were seeded into discs of JDBM, Ti, and cell culture plates with or without 15% extract and MgCl_2_ conditioning medium for 1 h and then stimulated with or without 1 μg/ml of LPS for another 24 h. According to previous reports, LPS could stimulate macrophages at 10 ng/ml−1 μg/ml, and the higher the inflammatory response macrophages were induced, the more effective the inhibition of the alloy became if it had anti-inflammatory capacity; 1 μg/ml of LPS, therefore, was selected in our study (36, 37). The pH value and magnesium ion concentration of supernatant from the groups were analyzed using a pH detector (PB-10, Sartorius, Germany) and inductively coupled plasma–atomic emission spectrometry (ICP-AES; PerkinElmer Optima 2000, USA), respectively. For evaluating cytotoxicity, the supernatants were tested using a lactate dehydrogenase (LDH) cytotoxicity assay kit (Beyotime, China) according to the protocol.

### Scanning Electron Microscopy

THP-1 cell-derived macrophages were seeded on the surface of JDBM and Ti discs for 1 h before the addition or not of LPS for 24 h and then fixed in 2.5% paraformaldehyde (PFA) for 40 min followed by gradient isopropanol dehydration (20, 40, 60, 80, 95, and 100%, 10 min each step). After being dried under vacuum, the surface of the samples was coated with gold. The samples were then observed by scanning electron microscopy (SEM) (SHINKKUVD MSP, Japan).

### Real-Time Quantitative PCR Analysis

THP-1 cell-derived macrophages were treated using the method as described above. The total RNA extractions and cDNA synthesis were performed using kits (TOYOBO, Japan). Bio-Rad C100 was employed for RT-qPCR analysis using SYBR green (TOYOBO, Japan). The levels of target genes were normalized to GAPDH, a housekeeping gene, for calculation using the 2^−ΔΔCT^ method. The primer sequences of genes are listed in Table S1.

### Enzyme-Linked Immunosorbent Assay

ELISA was carried out to determine the expression of pro-inflammatory cytokines (IL-6 and TNF). THP-1 cell-derived macrophages were treated with the same method described above. The level of cytokines in the supernatant was determined using an ELISA kit (DAKWE, China) according to the manufacturer's instructions.

### Western Blotting

Protein lysates extracted from cells were loaded into 10% or 12% sodium dodecyl sulfate–polyacrylamide gels for electrophoresis (SDS-PAGE). After that, proteins were transferred to polyvinylidene difluoride (PVDF) transfer membranes (Millipore, Billerica, USA). Primary antibodies, including MYD88, P65, P38, p-P38, ERK, p-ERK, JNK, p-JNK, p-IKK-α/β, IKK-α/β, p-IκBα, IκB AKT, p-AKT, AKT1, and p-AKT1 (CST, USA), were separately incubated overnight after blocking with 7% skim milk for 1 h. Then, secondary antibodies IRDye® 800CW goat anti-mouse IgG and IRDye® 800CW goat anti-rabbit IgG (Li-COR, USA) were incubated with the membranes for 1 h. Finally, all results were acquired by an infrared imaging system (Li-Cor Odyssey, Li-COR, USA). The data were analyzed by ImageJ software.

### Immunofluorescence and Flow Cytometry

The effects of extract and MgCl_2_ on NF-κB activity were evaluated with an NF-κB activation-nuclear translocation assay kit (Beyotime, China). THP-1 cell-derived macrophages were seeded into six-well plates with extract and MgCl_2_ for 1 h and then added with or without LPS for 30 min. After rinsing, fixation, and blocking, macrophages were incubated with p-65 primary antibody at 4°C overnight. Cells were subsequently incubated with cy3-conjugated secondary antibody for 1 h and then stained with DAPI for 5 min at room temperature. Finally, macrophages were visualized by fluorescence microscopy (DFC310, LECI, Germany).

For the intracellular ROS detection, cells were pretreated with extract or MgCl_2_ for 1 h and then stimulated with LPS for 1 h. Next, cells were stained using dichlorodihydrofluorescein diacetate (DCFH, Beyotime, China) according to the protocol. Finally, THP-1 cell-derived macrophages were harvested and analyzed by flow cytometry [fluorescence-activated cell sorting (FACS); Canto II, BD, USA] or directly visualized using a fluorescence microscope.

For the TLR-4 detection, cells were pretreated with extract or MgCl_2_ for 1 h before LPS stimulation for 24 h. Cells were washed with PBS and stained with TLR-4-PE (BioLegend, USA) for 30 min. After that, the results were analyzed with FACS.

The FACS data were processed using Flowjo 7.6 software.

### siRNA Transfection

TRPM7 siRNA was obtained from the Beijing Genomics Institute, China. THP-1 cell-derived macrophages were seeded into six-well plates for 12 h before transfection with siRNA and Lipofectamine 6000 (Beyotime, China) for another 48 h according to the instructions.

### Statistical Analysis

The statistical analyses were performed by using one-way ANOVA with Tukey's honestly significant difference (HSD) on SPSS software. All results were analyzed as means ± standard deviation (SD) and *P* < 0.05 was considered as statistical significance.

## Results

### Effects of JDBM, Extract, and MgCl2 on the Pro-Inflammatory Response of Lipopolysaccharide-Induced THP-1 Cell-Derived Macrophages

**Figure 1:**
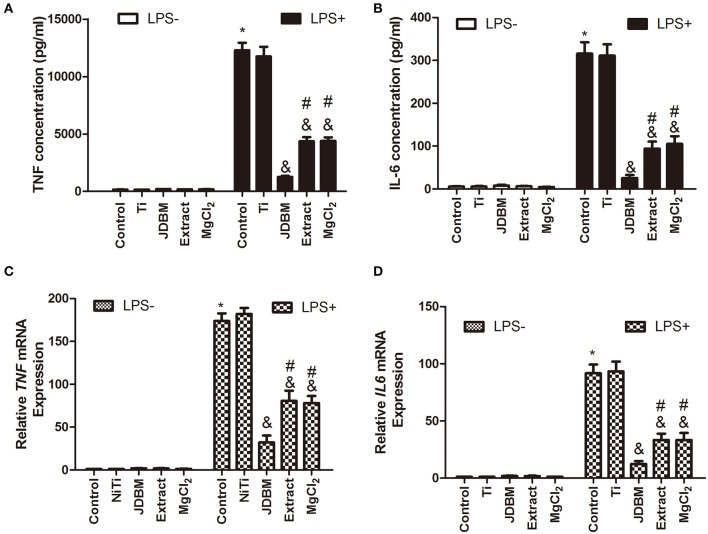
Effects of JDBM, extract, and MgCl_2_ on the expression of pro-inflammatory cytokines in THP-1 cell-derived macrophages. THP-1 cell-derived macrophages were cultured with JDBM, Ti, extract, and MgCl_2_ for 1 h prior to addition of LPS for another 6 or 24 h. The protein expression of TNF **(A)** and IL-6 **(B)** in supernatants was determined at 24 h by ELISA. The mRNA expression of *TNF* **(C)** and *IL-6* **(D)** was analyzed with qPCR at 6 h. ^&^*P* < 0.05 vs. LPS-induced control group; *P < 0.05 vs. control group; ^#^*P* < 0.05 vs. LPS + JDBM group. JDBM, Mg-2.1Nd-0.2Zn-0.5Zr alloy; LPS, lipopolysaccharide; TNF, tumor necrosis factor; IL, interleukin.

Because magnesium is the main degradable product of Mg-based alloys (38), we investigated the role of magnesium from JDBM on the anti-inflammatory response by setting up a 15% extract group and a similar magnesium concentration as a 7.5 mM MgCl_2_ group. The effects of JDBM, extract, and MgCl_2_ solution on the secretion of inflammatory cytokines were first investigated by ELISA and qPCR. JDBM, extract, and MgCl_2_ suppressed the expression of TNF-α and IL-6 in LPS-stimulated macrophages compared with the LPS control group at both the protein (Figures 1A,B) and the mRNA (Figures 1C,D) levels, but the Ti group did not have this effect. Notably, the expression of TNF and IL-6 in the JDBM group was significantly lower in the extract and the MgCl_2_ group. In addition, no significant difference was observed in all groups without LPS stimulation.

### Cytotoxicity and Cellular Morphology of THP-1 Cell-Derived Macrophages on JDBM and Ti Discs

**Figure 2:**
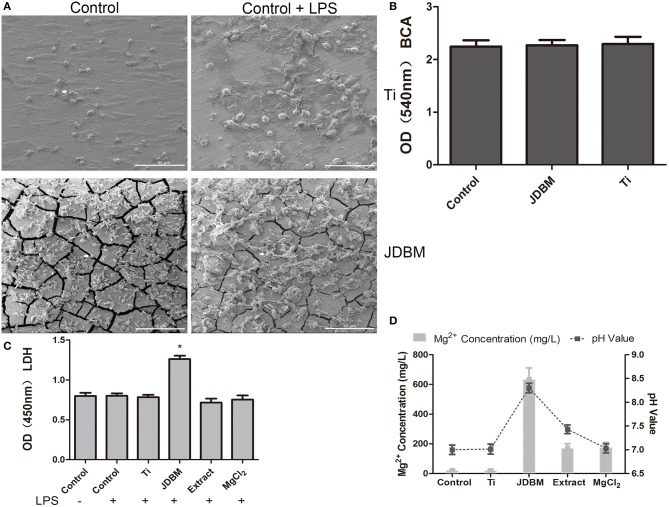
The characterization of the effects of JDBM, Ti, extract, and MgCl_2_ on the macrophages. **(A)** Macrophages were seeded on JDBM or Ti discs with or without LPS for 24 h after immersion in culture media for 24 h, and the cellular morphology of macrophages was visualized by SEM. Scale bar = 80 μm. **(B)** The JDBM, Ti, and cell culture plates (CCPs) were immersed in culture media for 24 h, and the protein concentration of supernatants was determined by the BCA assay. THP-1 cell-derived macrophages were precultured with JDBM, Ti, extract, and MgCl_2_ for 1 h prior to challenge with or without LPS for 24 h. The supernatants were harvested to analyze the cytotoxicity **(C)** and the pH value and Mg^2+^ concentration **(D)**. The representative images from three experiments are shown. *P < 0.05 vs. control group. JDBM, Mg–Nd–Zn–Zr alloy; LPS, lipopolysaccharide; SEM, scanning electron microscopy; BCA, bicinchoninic acid.

To further investigate the complex reasons for the anti-inflammatory capacity of JDBM, we evaluated the difference in protein adsorption among the Ti, JDBM, and CCP by BCA assay because of its importance as mentioned in *Materials Preparation*, whereas no significant difference was visualized between all groups (Figure 2B). Next, because macrophages with different cellular morphology could represent a pro-inflammatory or anti-inflammatory subset of cells, we further analyzed the cellular morphology of macrophages on the surface of JDBM and Ti discs (39). As shown in Figure 2A, macrophages showed a round type and a flat type in both JDBM and Ti, whereas, after LPS stimulation, they changed into an “omelet-like” type with spread pseudopodia. However, there was no significant distinction between cells of the JDBM and Ti groups under LPS stimulation or not. Moreover, we also found that JDBM caused visible cell damage compared with the control group, whereas the other groups had no significant cytotoxicity (Figure 2C). Furthermore, the ICP results showed that the magnesium concentration of JDBM was remarkably higher than that of the extract and the MgCl_2_ group, and the pH value of JDBM was also higher than that of the other groups, which indicated that alkalinity and the extremely high magnesium concentration of the environment might result in cytotoxicity (Figure 2D). In addition, we detected Zn < 0.5 ppm, Zr < 0.2 ppm, and Nd < 0 ppm of extract group compared with those of control group (Zn < 0.2 ppm, Zr < 0 ppm, and Nd < 0 ppm), suggesting that these element had no effects owing to extremely low concentration (data not shown). Together, the JDBM group had a better anti-inflammatory effect than the extract and the MgCl_2_ group because high magnesium ion levels are produced, causing alkalinity, thereby resulting in cytotoxicity, instead of changing cellular morphology.

### The Role of Magnesium in JDBM on Anti-inflammation of Lipopolysaccharide-Induced THP-1 Cell-Derived Macrophages

**Figure 3:**
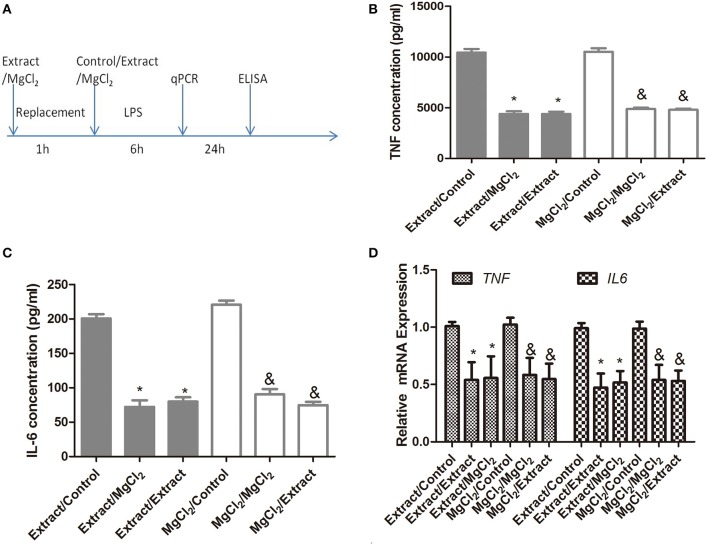
The reversible anti-inflammatory effects of extract and MgCl_2_ **(A)** THP-1 cell-derived macrophages were cultured with extract or MgCl_2_ for 1 h and replaced with extract, MgCl_2_, or normal media (control) in the presence of LPS for the indicated times. The protein expression of TNF **(B)** and IL-6 **(C)** in supernatants was determined at 24 h by ELISA. The mRNA expression of *TNF* **(C)** and *IL-6* **(D)** was analyzed with qPCR at 6 h. ^&^*P* < 0.05 vs. MgCl_2_/control group; *P < 0.05 vs. extract/control group. LPS, lipopolysaccharide; TNF, tumor necrosis factor; IL, interleukin.

Before the anti-inflammatory effects of extract and MgCl_2_ were proven, as shown in Figure 1, we further analyzed whether this effect of magnesium ion was reversible. Macrophages were treated with or without extract and MgCl_2_ for 1 h. Cells were washed, and LPS was added in the presence of control, extract, or MgCl_2_ solution for another 6 or 24 h. As shown in Figure 3, exposure to extract or MgCl_2_ before the LPS challenge had little influence on the production of IL-6 and TNF by macrophages at both the protein and RNA levels, suggesting that the anti-inflammatory effects of magnesium ion were reversible. These results also indicated that magnesium ion downregulated pro-inflammatory cytokines ahead of transcription.

### Effects of Extract and MgCl_2_ on the TLR-4 Pathway During the Lipopolysaccharide-Induced Inflammatory Response

**Figure 4:**
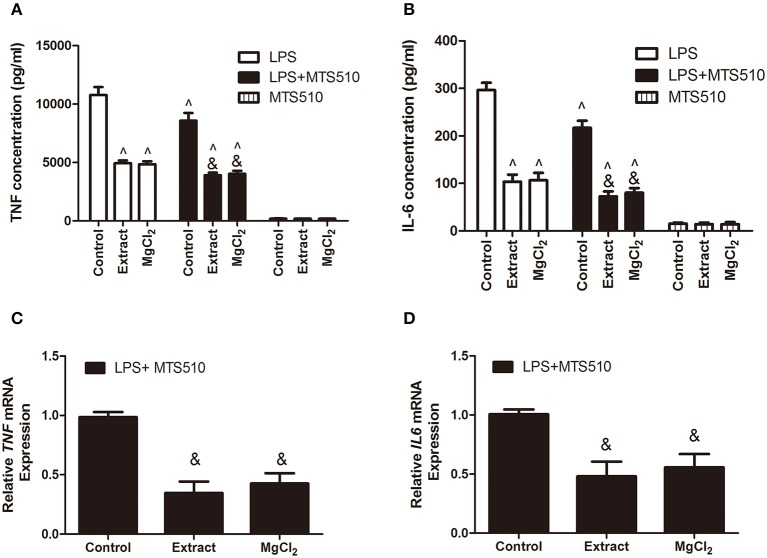
Effects of extract and MgCl_2_ on the expression of pro-inflammatory cytokines of THP-1 cell-derived macrophages through regulation of the TLR-4 pathway. THP-1 cell-derived macrophages were pretreated with anti-TLR-4 antibody (MTS510, 3 μg/ml) for 1 h prior to the addition of extract or MgCl_2_ for another 1 h and then stimulated with LPS for the indicated time. The protein expression of TNF **(A)** and IL-6 **(B)** in supernatant was measured by ELISA at 24 h. The mRNA expression of *TNF* **(C)** and *IL-6* **(D)** was analyzed with qPCR at 6 h. ^&^*P* < 0.05 vs. LPS + MTS510 control group; ^∧^*P* < 0.05 vs. LPS-induced control group. TLR, toll-like receptor; LPS, lipopolysaccharide; TNF, tumor necrosis factor; IL, interleukin.

**Figure 5:**
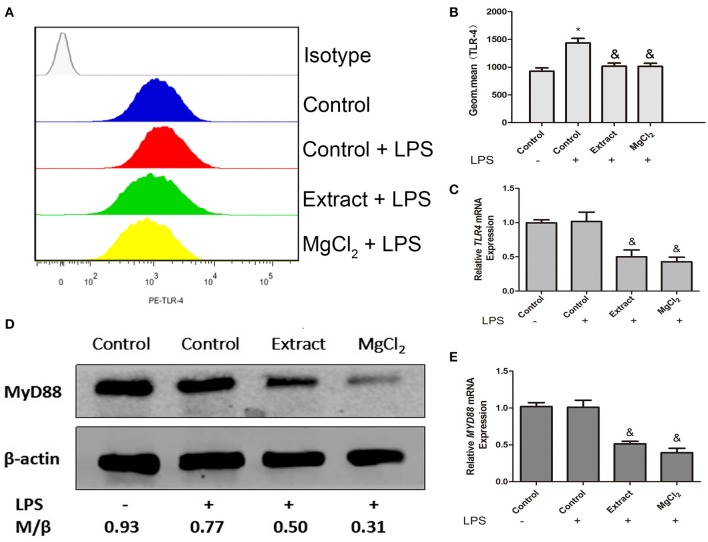
Effects of extract and MgCl_2_ on TL-4–MYD88-dependent signaling pathway in THP-1 cell-derived macrophages. THP-1 cell-derived macrophages were pretreated with MgCl_2_ or extract for 1 h prior to challenge with LPS for another 24 h. The expression of TLR-4 was analyzed by FACS **(A,B)**, and MYD88 was detected at the protein level using western blotting **(D)**. The expression of *TLR-4* and *MYD88* was measured at the mRNA level using real-time PCR **(C,E)**. The representative images from three experiments are shown. *P < 0.05 vs. control group; ^&^*P* < 0.05 vs. LPS-induced control group. LPS, lipopolysaccharide; FACS, fluorescence-activated cell sorting.

According to the above results, we next investigated the effects of extract and MgCl_2_ on the inflammatory signaling pathway. Because it was the first cascade of LPS binding to macrophage TLR-4 during the inflammatory process, it was necessary to examine whether extract and MgCl_2_ inhibited the LPS-induced inflammatory response via the TLR-4 receptor. An anti-TLR-4 monoclonal antibody (MTS510) was used to treat macrophages prior to stimulation with LPS. The results revealed that MTS510 with or without extract and MgCl_2_ depressed TNF and IL-6 release after LPS challenge. Of note, treatment of macrophages with a combination of MST510 and extract or MgCl_2_ synergistically inhibited IL-6 and TNF expression elicited by LPS compared with treatment with MTS510 alone (Figure 4). To further analyze how magnesium ion affected the TLR-4 signaling pathway, the effects of extract and MgCl_2_ on the expression of TLR-4 and MYD88 in LPS-induced macrophages were determined. As shown in Figure 5, both the extract and MgCl_2_ groups, compared with the LPS-induced control group, could reduce TLR-4 and MYD88 expression at both the RNA and protein levels. Altogether, these results showed that magnesium ion could downregulate the TL-4/MYD88 signaling pathway.

### Effects of Extract and MgCl2 on the Nuclear Factor-Kappa B and Mitogen-Activated Protein Kinase Pathway in Lipopolysaccharide-Induced THP-1 Cell-Derived Macrophages

**Figure 6:**
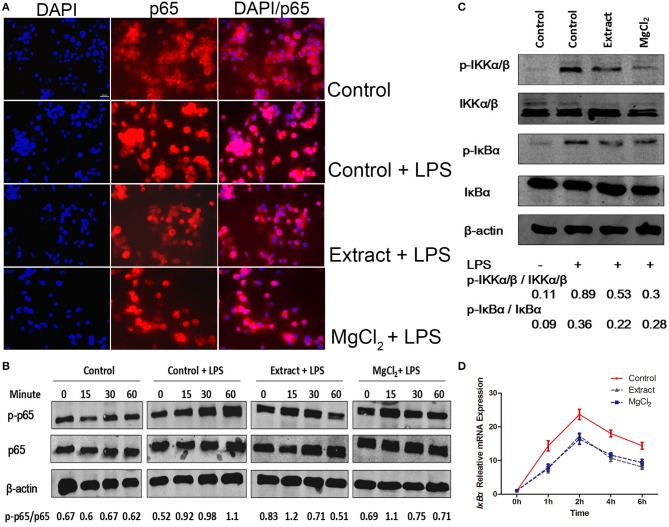
Effects of extract and MgCl_2_ on the NF-κB inflammatory signaling pathway in LPS-induced THP-1 cell-derived macrophages. Cells were pretreated with extract or MgCl_2_ for 1 h and then stimulated with LPS for 30 min. **(A)** Immunofluorescence images of control, LPS + control, MgCl_2_ + LPS, and extract + LPS groups. Scale bar = 20 μm. **(B)** The p65 activity in THP-1 cell-derived macrophages was determined at the indicated times by western blotting. **(C)** Cells were pretreated with extract or MgCl_2_ for 1 h and then stimulated with LPS for 30 min. The relative expression of p-IKβ-α/IKβ-α and p-IKK-α/β/IKK-α/β was determined by western blotting. **(D)** THP-1 cell-derived macrophages were pretreated with extract or MgCl_2_ and then stimulated with LPS at various time points. The increase in LPS-induced *I*κ*B-*α gene expression was in the presence of extract or MgCl_2_ analyzed by qPCR. The representative images from three experiments are shown. NF-κB, nuclear factor-kappa B; LPS, lipopolysaccharide.

**Figure 7:**
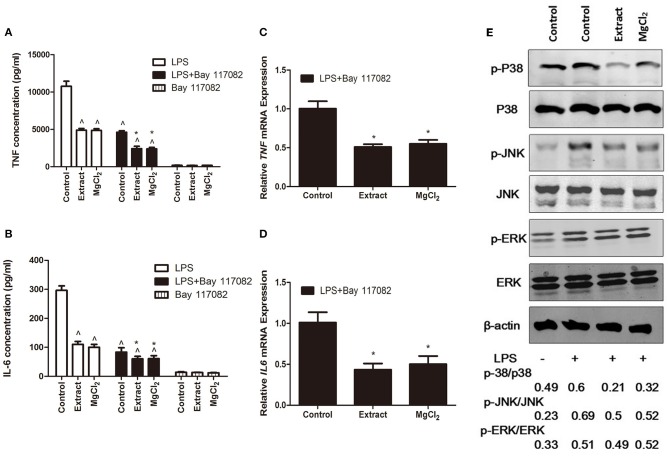
Effects of extract and MgCl_2_ on the MAPK inflammatory signaling pathway in LPS-induced THP-1 cell-derived macrophages. THP-1 cell-derived macrophages were pretreated with or without NF-κB inhibitor (Bay 117082, 5 μM) for 1 h prior to the addition of extract or MgCl_2_ for another 1 h and then stimulated with LPS for the indicated time. The protein expression of TNF **(A)** and IL-6 **(B)** in medium was measured at 24 h by ELISA. The mRNA expression of *TNF* **(C)** and *IL-6* **(D)** was analyzed at 6 h by qPCR. **(E)** THP-1 cell-derived macrophages were pretreated with extract or extract for 1 h and then stimulated with LPS for 45 min. The relative expression of p-P38/p38, p-ERK/ERK, and p-JNK/JNK was determined by western blotting. The representative images from three experiments are shown. *P < 0.05 vs. LPS + Bay 117082 control group; ^∧^*P* < 0.05 vs. LPS-induced control group. MAPK, mitogen-activated protein kinase; LPS, lipopolysaccharide; NF-κB, nuclear factor-kappa B; TNF, tumor necrosis factor; IL, interleukin.

NF-κB is a critical mediator downstream of the TLR-4 pathway to produce inflammatory cytokines; thus, we next evaluated NF-κB activity in the presence of extract or MgCl_2_ after LPS stimulation (40). As shown in Figure 6A, the P65 protein (NF-κB) was transferred into the nucleus from the cytosol in the presence of LPS, whereas extract and MgCl_2_ reversed the process. Furthermore, the results of western blotting also showed that both the extract and MgCl_2_ groups could decrease the phosphorylation of P65, compared with the LPS control group, in a time-dependent manner (Figure 6B). Moreover, the LPS-induced phosphorylation of IκBα, a repressor of NF-κB, and of IKK-α/β, a crucial upstream protein of NF-κB, was also significantly reversed in the extract and MgCl_2_ groups compared with the LPS control group (Figure 6C). In addition, the extract and MgCl_2_ groups, compared with the LPS control group, could attenuate the increasing trend of LPS-induced *I*κ*B*α expression at the mRNA level for 2 h (Figure 6D). To further comprehensively disclose the effects of magnesium on the TLR-4 pathway, an anti-NF-κB inhibitor (Bay 117082) was added to macrophages before the LPS challenge. As shown in Figures 7A–D, the groups of extract or MgCl_2_ with or without Bay 117082 could inhibit TNF and IL-6 release inflicted by LPS, compared with the LPS control group, at both the protein and mRNA levels. Interestingly, treatment of macrophages with a combination of Bay 117082 and extract or MgCl_2_ synergistically inhibited IL-6 and TNF expression by LPS challenge, compared with treatment with Bay 117082 alone, which indicates that there are other transcription factors (TFs) affected by magnesium. The MAPK pathway, including p38, ERK, and JNK, is another important pathway downstream of TLR-4 to regulate activator protein 1 (AP-1) translocation into the nucleus, thereby enhancing inflammatory cytokine expression (41). Thus, we next investigated the activity of MAPK in the presence of extract and MgCl_2_. As shown in Figure 7E, the phosphorylation of P38 and JNK stimulated by LPS was significantly reversed by extract and MgCl_2_, compared with the LPS control group, whereas the phosphorylation level of ERK was not significantly different. All these results suggested that magnesium ion was able to inactivate the NF-κB and MAPK signaling pathways to inhibit the TLR-4 signaling axis.

### Effects of Extract and MgCl_2_ on the Lipopolysaccharide-Induced Reactive Oxygen Species Production of THP-1 Cell-Derived Macrophages

**Figure 8:**
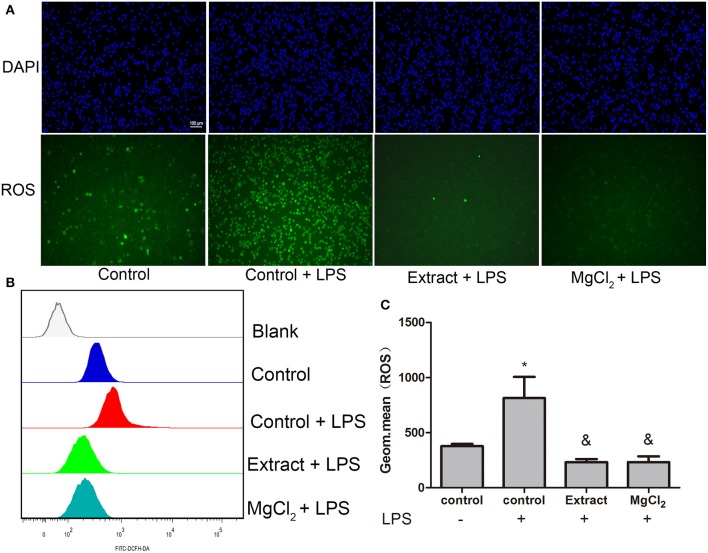
Effects of extract and MgCl_2_ on antioxidants in LPS-induced THP-1 cell-derived macrophages. Cells were pretreated with MgCl_2_ or extract for 1 h and then stimulated with LPS for 1 h. **(A)** The ROS level of THP-1 cell-derived macrophages was assayed by fluorescence microscopy using a DCFH probe. Scale bar = 100 μm. **(B,C)** The expression of intracellular ROS was also detected by FACS using a DCFH probe. The representative images from three experiments are shown. *P < 0.05 vs. control group; ^&^*P* < 0.05 vs. LPS-induced control group. LPS, lipopolysaccharide; ROS, reactive oxygen species; DCFH, dichlorodihydrofluorescein diacetate; FACS, fluorescence-activated cell sorting.

Previous research revealed that LPS could induce ROS production and that ROS further contributed to the enhancement of the TLR-4 pathway by interacting with NF-κB, which increased the inflammatory response (42). Therefore, the possible effect of magnesium on intracellular ROS expression was evaluated by FACS and fluorescence microscopy. As shown in Figure 8, extract and MgCl_2_ could effectively decrease ROS production inflicted by LPS compared with the LPS control group. These results indicated that magnesium ion might be a good ROS scavenger.

### The Role of TRPM7 on Inhibiting Inflammation of Extract and MgCl_2_

The above findings prompted us to explore how magnesium affected the TLR-4 signaling pathway. Because of the possibility that extracellular magnesium depressed LPS/TLR binding activity, macrophages were stimulated by LPS for 15 min to permit LPS/TLR-4 binding before extract or MgCl_2_ replacement with LPS, whereas the IL-6 and TNF expression in the presence of extract or MgCl_2_ was still decreased, compared with the LPS-induced control group at both the protein and mRNA levels (Figure S1). Together, these results supported the concept that magnesium affected inflammation by an intracellular molecular mechanism.

**Figure 9:**
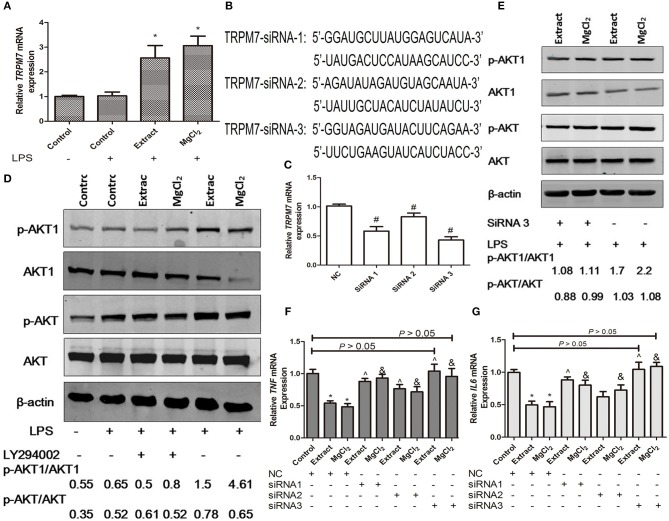
Effects of extract and MgCl_2_ on the TRPM7–PI3K–AKT1 anti-inflammatory signaling pathway in macrophages. **(A)** The mRNA expression of *TRPM7* in THP-1 cell-derived macrophages cultured with extract or MgCl_2_ for 1 h prior to the addition of LPS for 6 h was determined by qPCR. **(B,C)** THP-1 cell-derived macrophages were, respectively, transfected with TRPM7-siRNAs (TRPM7-siRNA1, TRPM7-siRNA2, and TRPM7-siRNA3) plasmids for 72 h, and the mRNA expression of *TRPM7* was determined by qPCR. **(D)** THP-1 cell-derived macrophages were pretreated with extract or MgCl_2_ in the presence or absence of LY294002 (10 mM) for 1 h prior to stimulation with LPS for 45 min. The relative expression of p-AKT/AKT and p-AKT1/AKT1 was determined by western blotting. **(E)** THP-1 cell-derived macrophages were transfected with TRPM7-siRNA3 or NC (no plasmid content) for 72 h and then incubated in the presence or absence of extract or MgCl_2_ for 1 h prior to stimulation with LPS for 45 min. The relative expression of p-AKT/AKT and p-AKT1/AKT1 was determined by western blotting. The transfected THP-1 cell-derived macrophages were pretreated with or without extract or MgCl_2_ for 1 h prior to stimulation with LPS for 6 h and the mRNA expression of *TNF* **(F)** and *IL-6* **(G)** was analyzed by qPCR. The representative images from three experiments are shown. *P < 0.05 vs. LPS control group or NC-control group; ^∧^*P* < 0.05 vs. NC-extract group; ^#^*p* < 0.05 vs. NC group; ^&^*P* < 0.05 vs. NC-MgCl_2_ group. LPS, lipopolysaccharide.

Next, we tried to investigate the possible role of TRPM7 during the anti-inflammatory process of magnesium in the LPS-stimulated macrophages. As shown in Figure 9A, the expression of TRPM7 in the presence of extract and MgCl_2_ was higher than that of the control group at the RNA level. Next, we transfected TRPM7-siRNA into macrophages, and the qPCR results showed that all of the siRNA could significantly suppress *TRPM7* expression, especially siRNA3 (Figures 9B,C). Although previous studies proved the antisense of siRNA at the protein level in other cells, the TRPM7 protein expression of siRNA transfected cells was not detected in this study, which might be a weakness (43, 44).

Then, the transfected macrophages were treated with extract or MgCl_2_ for 1 h before LPS stimulation for another 6 h, and qPCR was used to analyze the *TNF* and *IL-6* expression at the mRNA level. As shown in Figures 9F,G, after LPS stimulation, the *TNF* and *IL-6* expression of TRPM7 knockdown macrophages with extract or MgCl_2_ was significantly increased compared with that of macrophages in the presence of extract or MgCl_2_ alone. In particular, the siRNA3 group was not significantly different from the LPS control group (*P* > 0.05), which suggested that TRPM7 was a necessary factor during the anti-inflammatory process associated with magnesium. To further systematically disclose the role of TRPM7 in the anti-inflammatory response of magnesium ion, a PI3K inhibitor (LY24002) was added to macrophages prior to LPS challenge with or without extract or MgCl_2_, and then the activity of AKT and AKT1 was evaluated by western blotting. As shown in Figure 9D, the phosphorylation level of AKT in the LPS control, extract + LPS, and MgCl_2_ + LPS groups were higher than that of the control group. The phosphorylation level of AKT1 in the group of extract + LPS or MgCl_2_ + LPS was significantly increased compared with that of the LPS control group and control group, whereas the groups of extract + LY24002 + LPS and MgCl_2_ + LY24002 + LPS could decrease their phosphorylation compared with that of groups without LY24002, which suggested that magnesium inhibited inflammation by regulation of the PI3K/AKT1 pathway. Then, the relationship between the activity of AKT1 and TRPM7 was analyzed by western blotting and showed that the phosphorylation of AKT1 in LPS + extract + TRPM7-SiRNA3 or LPS + MgCl_2_ + TRPM7-SiRNA3 group was lower than that of LPS + extract or LPS + MgCl_2_ group, respectively, whereas the activity of AKT had no influence (Figure 9E). Together, these results showed that magnesium inhibited the inflammatory response of macrophages through regulation of the TRPM7–PI3K–AKT1 pathway.

## Discussion

Although Mg-based alloys are a promising biomaterial for the future, local inflammation caused by FBR, infection, or surgery remains a concern (45). The present study systematically analyzed the potential mechanisms of the degradable products of JDBM in the LPS induction of the pro-inflammatory response of macrophages. We found that magnesium degradable products played a critical factor in the anti-inflammation effect of a Mg-based alloy. It effectively inhibited pro-inflammatory cytokine release induced by the TLR-4 pathway through activating the TRPM7–PI3K–AKT1 pathway.

Rapid corrosion of a Mg-based alloy would have many side effects, including massive accumulation of these products, thereby resulting in high magnesium concentration and alkalinity, which in turn deteriorated the local physical condition (7). Evidence has shown that overrated magnesium would impair cell viability (46, 47). Our previous study also showed that above 50%, extract would result in cytotoxicity of macrophages, and this was partly the reason for anti-inflammation effects of the Mg-based alloy; besides, 20% extract could trigger inflammatory response without LPS stimulation owing to possible high osmotic pressure, although it was also able to inhibit inflammation after LPS stimulation (32). Therefore, it was reasonable to describe why JDBM inhibited inflammation “better” than 15% extract, and the reason why we selected 15% extract in our study was it will not trigger inflammation but has anti-inflammation effects after LPS stimulation, which offered a suitable dilution time of extract in the future research. Furthermore, Li et al. found that Raw 264.7 macrophages were round and flat on the Ti alloy, whereas the cells in the Mg-doped Ti alloy were more elongated and less flattened, which demonstrated that magnesium promoted macrophage polarization (48). However, in our experiments, we did not find this process; and the possible causes, we speculated, might be using a different macrophage cell line and adding LPS to mimic an infectious environment.

Previous data have revealed that magnesium suppressed the inflammatory response by decreasing the activity of the NF-κB pathway in various cells (17, 49, 50). Consistently, our results found that magnesium, as a major degradable product of the Mg-based alloy, suppressed the inflammatory response by downregulation of the TLR-4–MYD88–NF-κB signaling pathway during the LPS stimulation. Of note, the expression TLR-4 and MYD88 of the extract and MgCl_2_ was lower than that of negative control group at the mRNA level, which indicated that magnesium could first affect transcription. We also found that degradable product of a Mg-based alloy inhibited LPS-induced ROS production in macrophages, which in turn alleviated the activation of the NF-κB pathway, even though the potential mechanism required further exploration. Recently, Yan et al. reported that magnesium could inhibit the immune response by downregulation of all members of the MAPK pathways (51). However, other studies found that magnesium could attenuate CoCl_2_-induced neuronal cell death by activating the ERK1/2 pathway and could inhibit the calcification of extracellular matrix, thereby protecting articular cartilage through ERK/autophagy pathway (52, 53). Interestingly, our results confirmed that magnesium could effectively decrease the activity of the p38 and JNK pathways induced by LPS but not the ERK1/2 pathway. In that, the ERK signal pathway regulated the cell growth, differentiation, migration, and so on rather than inflammation (54); we speculated that magnesium could active ERK1/2 protein during the LPS stimulation, although the precise molecular study is for elucidation in the future.

It had been reported that the PI3K/AKT pathway played a crucial role in preserving the integrity of the immune system (55). For example, Schabbauer et al. revealed that PI3K/AKT activation significantly enhanced endogenous anti-inflammation capacity (56). Of note, Su et al. found that PI3K/AKT activity was a crucial molecular mechanism underlying the anti-inflammation effects of MgSO_4_ during the LPS stimulation (57). Our study showed the extract, MgCl_2_, and LPS, compared with control, could activate AKT; however, the activity of AKT1, a subfamily AKT, was increased by extract and MgCl_2_ compared with the control group but not LPS. Given a previous study showing that AKT1 was an important mediator to promote macrophage polarization to an M2 type that had an anti-inflammatory effect (58), AKT1 reasonably played a more important factor than AKT in the anti-inflammatory capacity of magnesium. Also, Zhang et al. reported that TRPM7 could prevent magnesium ion movement into the cytoplasm to enhance expression of neuronal calcitonin gene-related polypeptide-a (CGRP) in both the peripheral cortex of the femur and the ipsilateral dorsal root ganglia (59). Consistently, our results also demonstrated that magnesium did not act as an anti-inflammation agent until entry into the cytoplasm, and notably, this process relied on TRPM7 regulation. Additionally, Zhang et al. found that magnesium could regulate the osteoinduction of human osteoblasts by the TRPM7–PI3K–AKT pathway (25). Our results showed that AKT1 was a major downstream of the TRPM7-PI3K pathway instead of AKT during the anti-inflammation of magnesium in the LPS-stimulated macrophages. Altogether, our study suggested that the degradable products of a Mg-based alloy limited the inflammation of macrophages via the TRPM7–PI3K–AKT1 signaling axis.

Although in this study we first showed that the degradation products of a Mg-based alloy exhibited an anti-inflammatory capacity through mediating the TRPM7–PI3K–AKT1 pathway, the rest of the TRPM families and anti-inflammatory signaling pathways have not been studied (60). Because THP-1 cell-derived macrophages were just a proxy for primary macrophages, it was a major limitation in our study, which needed a primary mouse or human macrophage to confirm the results in the future. Additionally, the related *in vivo* experiments should be implemented, and the effects of a Mg-based alloy on other TLRs need to be studied. Altogether, these findings provided some good evidence of Mg-based alloy application in infectious patients with conditions such as sepsis.

## Conclusion

**Figure 10:**
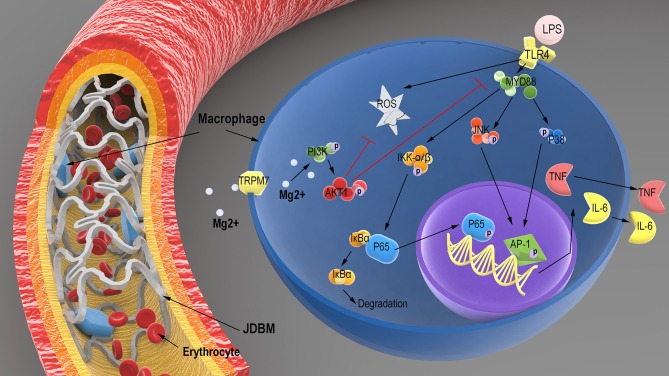
Schematic illustration of the potential regulatory mechanism of LPS-induced inflammatory responses in macrophages treated with the degradation products of JDBM. After JDBM cardiovascular stent was implanted, macrophages would adhere to the surface of stent owing to the FBR. The magnesium from the degradation products of the JDBM passed into the cytoplasm of macrophages through the TRPM7 channel to activate the PI3K–AKT1 signaling pathway and scavenged intracellular ROS to prevent the inflammatory response based on the LPS-induced activation of the TLR-4–MYD88–NF-κB/MAPK signaling pathway, which show good anti-inflammatory effects of Mg-based alloy. LPS, lipopolysaccharide; JDBM, Mg–Nd–Zn–Zr alloy; FBR, foreign body response; ROS, reactive oxygen species.

In this study, the degradable products of JDBM could effectively limit the inflammatory response by THP-1 cell-derived macrophages and might relieve FBR during implantation. We confirmed that magnesium from degradable products was a major factor in the anti-inflammatory process of JDBM. We found that intracellular magnesium could decrease the activity of the TLR-4–MYD88–NF-κB/MAPK signaling pathway and LPS-induced ROS expression, which depend on TRPM7 of THP-1 cell-derived macrophages regulating extracellular magnesium entrance, thereby activating the PI3K–AKT1 pathway to mediate the above pathway, as shown in Figure 10. Thus, our results provided a new mechanism for the anti-inflammatory capacity of Mg-based alloys, which should be taken into account prior to clinical application.

## Data Availability Statement

All datasets generated for this study are included in the article/Supplementary Material.

## Author Contributions

All authors listed have made a substantial, direct and intellectual contribution to the work, and approved it for publication.

### Conflict of Interest

The authors declare that the research was conducted in the absence of any commercial or financial relationships that could be construed as a potential conflict of interest.

## Supplementary Material

Figure S1 Role of the intracellular magnesium from Extracts or MgCl_2_ in the anti-inflammatory response. **(A)** THP-1 cell-derived macrophages were stimulated with LPS for 15 min prior to the addition of Extracts or MgCl_2_ for the indicated times. The protein expression of TNF **(B)** and IL-6 **(C)** in supernatants was measured at 24 h by ELISA. The mRNA expression of *TNF* and *IL-6* **(D)** was analyzed with qPCR at 6 h. ^&^*P* < 0.05 vs. control group.

Table S1 Primers used for real-time PCR.
