# Supplementary figures and images for: A Biodegradable Mg-Based Alloy Inhibited the Inflammatory Response of THP-1 Cell-Derived Macrophages Through the TRPM7–PI3K–AKT1 Signaling Axis
Source: Front Immunol. 2019 Dec 3;10:2798. doi: 10.3389/fimmu.2019.02798 (PMC6902094; doi:10.3389/fimmu.2019.02798)

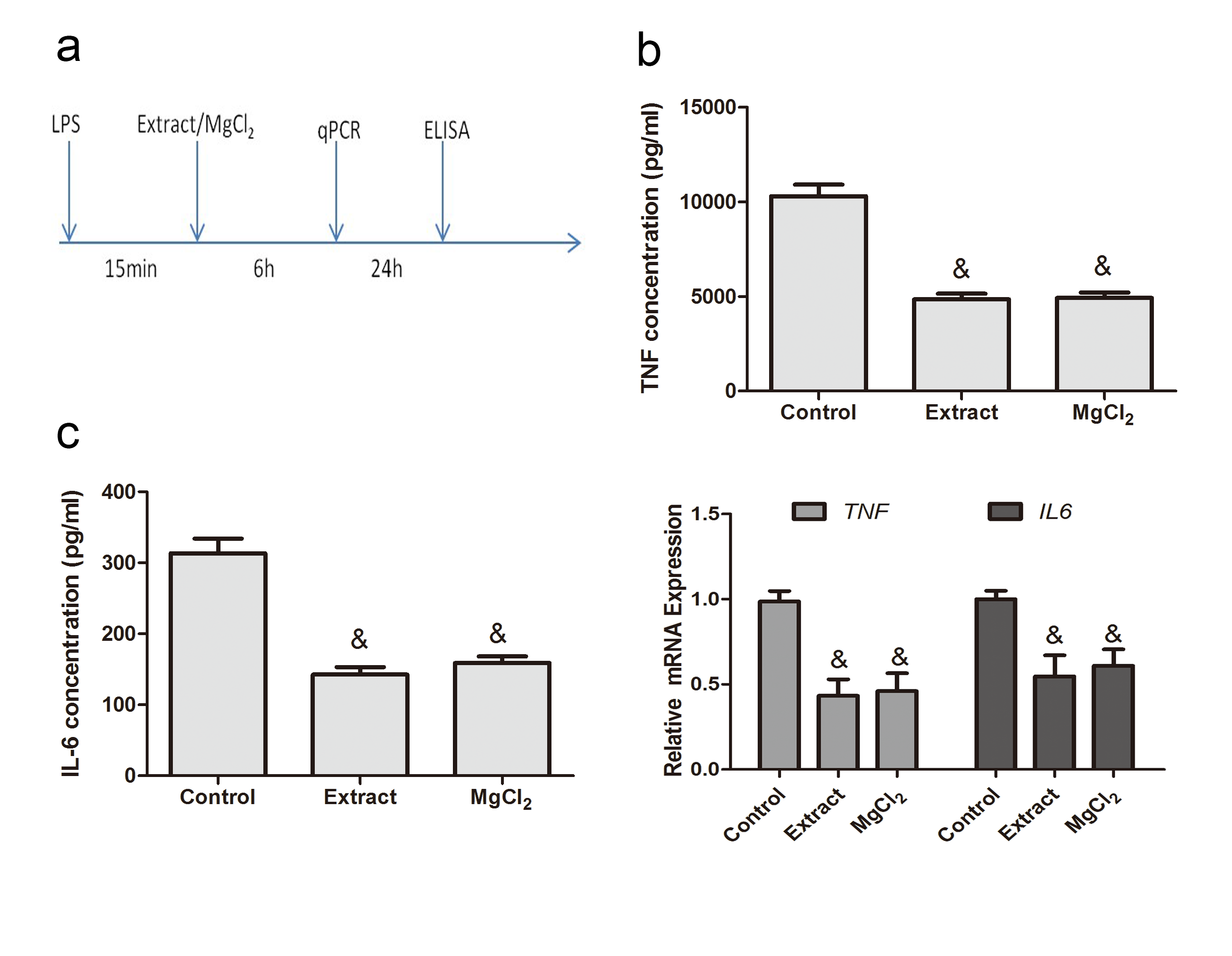

Supplement: Figure S1 — Role of the intracellular magnesium from Extracts or MgCl2 in the anti-inflammatory response. (A) THP-1 cell-derived macrophages were stimulated with LPS for 15 min prior to the addition of Extracts or MgCl2 for the indicated times. The protein expression of TNF (B) and IL-6 (C) in supernatants was measured at 24 h by ELISA. The mRNA expression of TNF and IL-6 (D) was analyzed with qPCR at 6 h. &P < 0.05 vs. control group. [file Image_1.TIF]
