# Supplementary material for: A Biodegradable Mg-Based Alloy Inhibited the Inflammatory Response of THP-1 Cell-Derived Macrophages Through the TRPM7–PI3K–AKT1 Signaling Axis
Source: Front Immunol. 2019 Dec 3;10:2798. doi: 10.3389/fimmu.2019.02798 (PMC6902094; doi:10.3389/fimmu.2019.02798)
Supplement: Table S1 — Primers used for real-time PCR. [file Table_1.DOCX]

Supplementary table 1. Primers used for real-time PCR

| Genes | Forward (5’-3’) | Reverse(5’-3’) |
| --- | --- | --- |
| *GAPDH* | GGAGAAGGCTGGGGCTCAT | TGATGGCATGGACTGTGGTC |
| *IκBα* | AAGTGATCCGCAGGTGAAG | TGCTGCAGGTTGTTCTGGAA |
| *MYD88* | GGCTGCTCTCAACATGCGA | AGACCTGTCCCTGAACCCTAT |
| *TLR4* | AGACCTGTCCCTGAACCCTAT | CGATGGACTTCTAAACCAGCCA |
| *TNF* | GTGATCGGCCCCCAGAGGGA | CACGCCATTGGCCAGGAGGG |
| *IL6* | CCACTCACCTCTTCAGAAC | CTTTGCTGCTTTCACACAT |
| *TRPM7* | CCCTACCGACCAAAGATTGA | CTTCATAAGGCAAGCCCAAA |
